# Graphene Family Nanomaterials: Properties and Potential Applications in Dentistry

**DOI:** 10.1155/2018/1539678

**Published:** 2018-12-09

**Authors:** Ziyu Ge, Luming Yang, Fang Xiao, Yani Wu, Tingting Yu, Jing Chen, Jiexin Lin, Yanzhen Zhang

**Affiliations:** ^1^Department of General Dentistry, The Second Affiliated Hospital, Zhejiang University School of Medicine, 310052, China; ^2^Zhejiang University, 310058, China

## Abstract

Graphene family nanomaterials, with superior mechanical, chemical, and biological properties, have grabbed appreciable attention on the path of researches seeking new materials for future biomedical applications. Although potential applications of graphene had been highly reviewed in other fields of medicine, especially for their antibacterial properties and tissue regenerative capacities,* in vivo* and* in vitro* studies related to dentistry are very limited. Therefore, based on current knowledge and latest progress, this article aimed to present the recent achievements and provide a comprehensive literature review on potential applications of graphene that could be translated into clinical reality in dentistry.

## 1. Introduction

Oral cavity is an extremely demanding setting. Dental materials when placed within oral cavity are fully contacted with saliva, gingival crevicular fluid, and water. At the same time, it is exposed to high temperature, masticatory forces, and variety of abrasion causing mechanical failures and overtime requiring restoration replacement with extra cost. Furthermore, most dental materials are in intimate contact with oral tissue for a long time; they must be noncytotoxic and biocompatible for them to have a harmonious interaction with host while performing desired functions. Therefore, there is always a huge interest and strong trend in continuous development of dental materials with improving properties.

**Figure 1:**
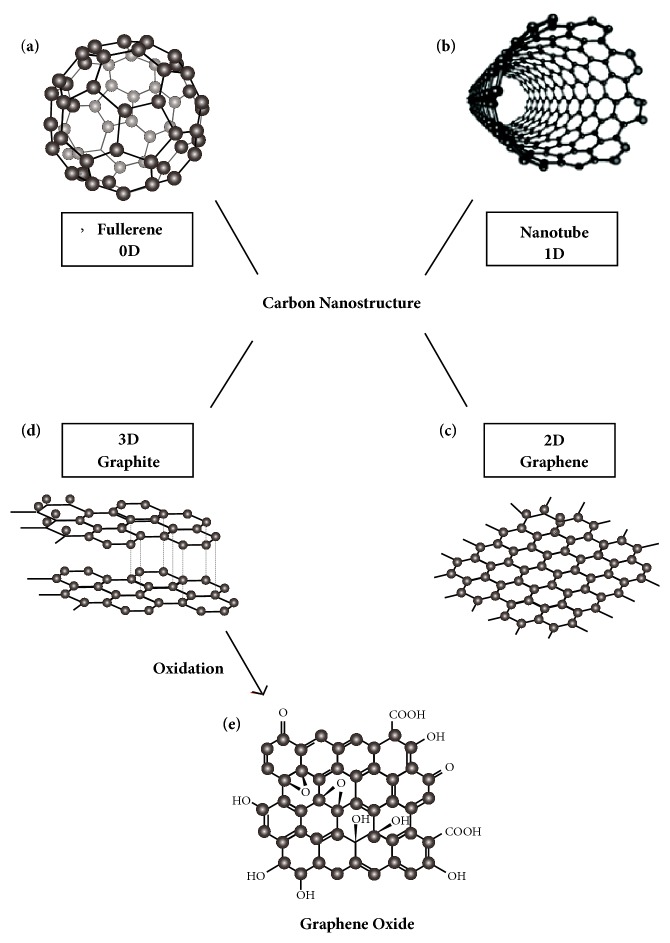
Different allotropes of carbon nanostructure: (a) 0D Fullerenes; (b) 1D Carbon Nanotubes; (c) 2D Graphene; (d) 3D Graphite. (e) Graphene Oxide can be synthesized through oxidation of graphite, with common method called Hummers method.

**Figure 2:**
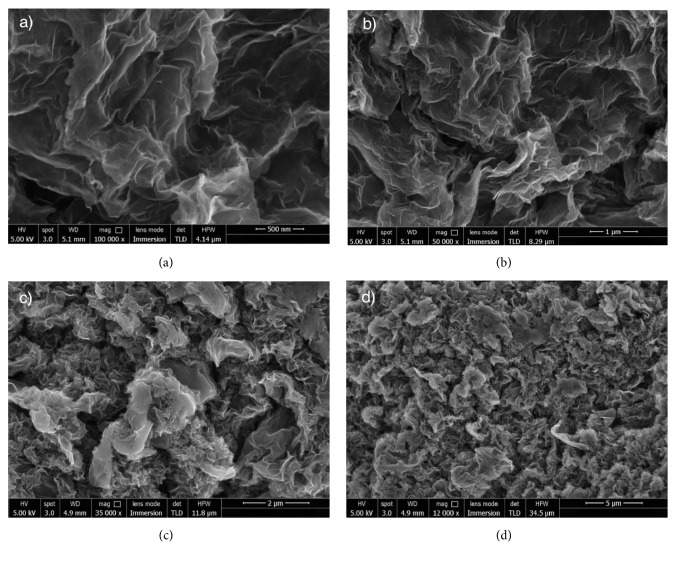
Graphene under scanning electron microscope (SEM) at (a) 100000× magnification, (b) 50000× magnification, (c) 35000× magnification, and (d) 12000× magnification.

Nanotechnology, “the manufacturing technology of the 21st century”, is an art of manipulating matter on a scale of less than 100nm to create numerous materials with various properties and functions. Over the past decades, with the discovery of fullerene in 1985 and carbon nanotubes in 1991, carbon based nanomaterials have been merited on the scientific stage (see Figure 1). Graphene is a 2D single layer of sp^2^ hybridized carbon atoms with hexagonal packed configuration (see Figure 2). The in-depth investigation of graphene conducted by Andre Geim and Konstantin Novoselov in 2004 has proven that graphene was the building block for all graphitic carbon materials such as graphite, diamond, nanoribbons, CNTs, and fullerenes. Moreover, it possesses exceptional physicochemical, optical, and mechanical properties. Since then, research efforts have been focused on excavating its potential applications including various biomedical applications such as drug delivery carriers [1], imaging agents [2], biosensors [3], bimolecular analysis, and tissue engineering scaffolds [4].

Graphene family nanomaterials (GFNs) include ultrathin graphite, few-layer graphene (FLG), graphene oxide (GO; from monolayer to few layers), reduced graphene oxide (rGO), and graphene nanosheets (GNS) [5]. They differ from each other in terms of surface properties, number of layers, and size [6]. Among other members of graphene family nanomaterial, graphene oxide (GO) is one of the most important chemical graphene derivatives which could be produced through energetic oxidation of graphite through Hummers method using oxidative agents. GO possessed a variety of chemically reactive functional groups on its surface, which facilitate connection with various materials including polymers, biomolecules, DNA, and proteins [7]. The large interactive aromatic surface area of GO is at least an order of magnitude higher compared with other nanomaterials endows it with high drug loading capacity [8]. Reduced graphene oxide (rGO) can be obtained by chemically, thermally, or electrochemically reducing graphene oxide, which possesses heterogeneous electron-transfer properties [9]. Fluorinated graphene (FG) is an uprising member in the graphene family. FG has favorable biocompatibility, exhibiting a neuroinductive effect via spontaneous cell polarization and enhancing adhesion and proliferation of mesenchymal cells providing scaffold for their growth [1].

Although the developments and researches of graphene-based biomaterials related to dentistry are still at infancy, their unique properties and their abilities to functionalize alone or combined with biomaterials offer several opportunities in possible clinical applications. In this review, we intended to provide readers with an overview of the potential applications of graphene correlated to dentistry. Their biocompatibility aspect and antibiotic properties were briefly discussed. Perspectives related to graphene-based technologies aimed at oral care are presented and organized by different fields of dentistry.

## 2. Biocompatibility

The first aspect to consider in the introduction of a new biomedical material is its biocompatibility. For a safer development of graphene-based nanomaterial, it is necessary to understand the interaction of graphene and their derivatives with living systems and their toxicity* in vivo* and* in vitro* [10].

Accumulating evidences have suggested that cytotoxicity of GFNs can not be generalized as it depends on various factors including their morphology (size, shape, and sharp edges), surface charge, surface functionalization, dispensability, state of aggregation, number of layers, purity, and methods of synthesis [11]. This is because different morphology, shape, and size of GFNs could influence their cellular uptake characteristics; moreover, distinctive functional groups on the surface can alter their interactions with proteins, biomolecules, and micronutrients. In relation to the concentration of GFNs on toxicity, many studies suggested that 50ug/ ml might be a toxicity threshold for GO on normal mammalian cells. Concentration higher than 50ug/ml might harm human fibroblast cells and T lymphocytes [7, 12]. Lateral size of GFN may determine the location of accumulation and amount of toxicity uptake by different organs in human body as nanoparticles with sizes <100nm can enter cell, <40nm can enter nucleus, and <35nm can cross blood brain barrier [13]. Surface structure, such as wettability, also plays a role in GFN's toxicity. Graphene is recognized as a hydrophobic material while GO is slightly hydrophilic due to the presence of oxygen containing functional groups on its basal plane [14]. In general, compared to hydrophobic ones, hydrophilic (more oxidized) graphene nanoparticles may be more cytocompatible as they tend to form a stable colloid dispersion and avoid aggregation, and, therefore, could be easier internalized and excreted from the application site [15]. GFNs' purity also deserves attention because sometimes contaminating metal may account for toxicity reaction in cells rather than GFNs themselves. Traditionally prepared GO often contains high levels of Mn^2+^ and Fe^2+^, which are highly mutagenic to cells leading to high levels of cytotoxicity and random scission of DNA [16]. Greener exfoliating methods suggested by Peng et al. were able to produce a high purity GO containing much lower Mn^2+^ and Fe^2+^ and consequently significantly lowered the cytotoxicity of GFNs [17]. Therefore, choosing a high purity GFNs synthesis method is a vital step toward safer bioapplications.

The exact mechanism underlying the toxicity of GFNs remains obscure; several possible toxicity mechanisms of GFNs were proposed. Oxidative stress is suggested to be the main cause as the elevated ROS level may oxidize various molecules including DNA, lipids, and proteins inducing apoptosis or necrosis [18]. GFNs may also cause cell necrosis by directly influencing cell mitochondrial activity through induction of mitochondrial membrane potential dissipation, which subsequently increases the generation of intracellular ROS and triggers apoptosis by activating the mitochondrial pathway [19]. For instance, GO with lower degree of oxidation possesses more free electrons facilitates more OH production from H_2_O_2_. The formation of OH and the cytochrome c/H_2_O_2_ electron-transfer system could enhance oxidative and thermal stress to impair the mitochondrial respiration system and eventually impose stronger oxidative damage on normal cells displaying stronger toxicity [20].

Studies focused on the cytotoxicity of GFNs in oral setting are very limited. A study conducted by Olteanu et al. assessed the cytotoxic potential of GO, thermally reduced graphene oxide (TRGO) and Nitrogen doped graphene (N–Gr), on human dental follicle stem cells. The result showed that GFNs, especially GO, increased the intracellular ROS generation in a concentration and time-dependent manner. At high concentration (40 ug /mL), cells viability was reduced and mitochondria membrane potential was altered. While, at low concentrations (4 ug/mL), they exhibited a good safety profile providing high antioxidant defense. Their authors concluded that, among these three investigated graphenes, GO exhibited the lowest levels of cytotoxicity and induced least amount of damage to human dental follicle stem cells [21].

Nevertheless, lack of consensus is reflected in* in vivo *studies as concentration and variations of GFNs tested can significantly change the toxicity outcome, not a conclusive answer can suit them all [22]. Thus, biosafety constraints, especially targeting dental tissue, should be solved to translate GFNs onto clinical applications.

## 3. Antibacterial Effect of Graphene

Interestingly, because of GNFs' versatility, their usage as potential antimicrobial agents has gained substantial interest in the field of nanomedicine [23–26]. However, the antibacterial effect of GFNs have reported to be controversial as the effect is also highly determined by size, shape, stability, and distribution [27] and the underlying experimental designs remained inconsistent [28].

A thorough understanding of the antimicrobial mechanism is still in its infancy, but, with an increasing number of investigations on the antimicrobial activities of GRNs, three predominate mechanisms were proposed. Physical damage is induced by blade like graphene materials piercing through the microbial cellular membrane causing leakage of intracellular substance leading to cell death. Wrapping and photothermal ablation mechanism could also provoke bacterial cell damage by enclosing the bacterial cells, providing an unique flexible barrier to isolate bacteria growth medium, inhibiting bacteria proliferation, and decreasing microbial metabolic activity and cell viability. Chemical effect is primary oxidative stress mediated with production of ROS as excessive intracellular ROS accumulation could cause intracellular protein inactivation, lipid peroxidation, and dysfunction of the mitochondria, which lead to gradual disintegration of cell membrane and eventual cell death [29]. There had also been researches theorizing that the antibacterial activity of graphene on metal substrate involved electron transfer from the bacterial membrane, producing ROS independent oxidative stress to the microbial membrane, interrupting electron transport in respiratory chain, and leading to destruction of microbial integrity and cell death [30]. A study conducted by Dellieu et al. tested the growth of* Escherichia coli* and* Streptococcus mutans* on gold and copper substrate in contact with CVD graphene and the result showed that the facile transfer of electrons from microbial membranes to graphene played no role on bacterial viability, denying the influence of metal substrates' conductive character on the antibacterial activity of CVD graphene [31].

Oral cavity is a complex ecosystem, forming structurally and functionally organized dental biofilm embedded in a matrix of polymers of host and bacterial origins. Of clinical relevance is the fact that imbalanced microbial homeostasis in dental biofilms is associated with etiology of dental diseases; for this reason, research efforts have been made to target putative pathogens by antimicrobial or antiadhesive strategies to prevent disease initiation and progression [32]. Although studies have established the antimicrobial activity of GNF against several bacteria such as* Escherichia coli*,* Streptococcus aureus*,* Klebsiella *sp., and* Pseudomonas aeruginosa*, only few studies focused on oral pathogens.* Streptococcus mutans* is the primary gram-positive facultative anaerobic bacteria involved in caries formation while* Porphyromonas gingivalis* and* Fusobacterium nucleatum *are Gram-negative anaerobic bacteria associated with periodontitis and root canal infection.

A study conducted by He et al. evaluated the antibacterial activity of GO nanosheets against these three common types of bacteria and found that GO nanosheets were highly effective in inhibiting the growth of dental pathogens. At GO concentration 40*μ*g/mL, bacterial growth of* P. gingivalis* and* F. nucleatum* was inhibited, while, at concentration 80 *μ*g/mL, GO absolutely killed all* S. mutans* [33]. This could be explained by the difference of resistance toward oxidative stress generated by GO between anaerobic and facultative anaerobic bacteria, in which GO nanosheets were more bactericidal against obligate anaerobic bacteria. It was also observed in TEM images that GO nanosheets can insert or cut through the cell membranes of bacteria and extract large amounts of phospholipids, mechanically destruct cell membrane integrity, and cause leakage of intracellular substances.

Another graphene derivative, graphene nanoplatelet, produced via thermal exfoliation of graphite intercalation compound, was also investigated for their antibacterial properties against* S. mutans*. Scanning electron microscopy analysis revealed that there is a strong mechanical interaction between cells and GNPs, firstly involving cell trapping and consequent shrinking and secondly involving piercing through soft cell wall with sharp edges of GNP flakes which eventually killed the planktonic form of* S. mutans* [34]. CVA-grown graphene also showed disruption of proliferation and formation of biofilm formed by* S. mutans* and* E. faecalis*, which infer to be due to the surface properties not electron diffusion of graphene material [35]. These research findings suggested that GNPs can be an effective dental material for controlling* S. mutans* and, consequently, caries, further proving the potential graphene hold for biomedical applications

Graphene, when combined with other compounds, showed improved synergistic antimicrobial, antibiofilm, and antiadherence activities against oral pathogens. Zinc oxide nanoparticles have been widely used in biomedical applications for its superior bactericidal effect, but its aggregation properties producing toxicity to mammalian cells have been hindering its use. When graphene material is synthesized with zinc oxide producing graphene/zinc oxide (GZNC) nanocomposite, it not only causes a much lower toxicity, but also forms a unique nanointerface to interact with microbes as compared to ZnO alone. It was observed that GZNC could decrease the synthesis of EPS, one of the key virulence factors of cardiogenicity, and reduce the amount of insoluble glucans, which influences biofilm formation by disturbing its physical integrity and stability, significantly reducing the biofilm and cariogenic properties* S. mutans *[36].

These antibacterial properties of GFNs could be very beneficial when integrating into biomaterials for potential clinical application. For instance, surgical sutures may be one of the most widely used medical adjunct nowadays and a good suture material should not only possess good mechanical properties, but also the antimicrobial ability to prevent breeding of bacteria. It was recently suggested that compared with conventional polyvinyl alcohol (PVA) fiber which has no antimicrobial property, PVA matrix dispersed with mechanically exfoliated graphene (MEG) showed high antibacterial effect toward gram-positive bacteria, thereby efficaciously accelerated the healing of wound, making PVA/MEG nanocomposite fiber a promising new candidate for surgical suture [37].

Studies on graphene-based materials for* in vivo* bactericidal applications are still at initial stage; whether graphene has long-term and broad-spectrum antibacterial effects stay debatable. A recent study suggested that GO is not an intrinsic antimicrobial material but a general growth enhancer that can act as a biofilm that allows bacterial attachment and proliferation [38]. Nevertheless, GFNs are still a potential antibacterial agent that are easy to obtain, cheaper, and capable of providing support to disperse and stabilize various nanomaterials synergistically yielding high antibacterial activities [39]. Additional in-depth experimental studies, especially toward dental pathogen, need to be introduced to further analyze the interaction between GFNs and biosystems for future clinical applications.

## 4. Potential Applications of Graphene in Tissue Engineering

Tissue loss due to trauma, disease, or congenital abnormality is a major healthcare concern worldwide. The ongoing challenge of dental treatment is to restore missing teeth with their periodontal structure [40]. There has been an evolution from the use of materials to simply replace diseased tissue, to that of utilizing specific biomaterials, which will nurture and regenerate a functionally and structurally acceptable tissue [41]. It is inevitable that tissue regeneration research topic is growing quickly in the clinical fields. Experimental development of stem cells together with their supporting biomaterials is a fundamental component of tissue engineering research [42].

Scaffolds play a significant role in tissue engineering. Either in the absence or presence of chemical inducers and growth factors, it should be optimally designed to provide a biocompatible three-dimensional environment that can not only mechanically ‘support' and ‘guide' bone regeneration, but also ‘stimulate' proliferation and differentiation of stem cells into their specific tissue lineage [43]. So far, most artificial biomaterials in the markets lack tissue inductive activities, which means that fast healings and functional reconstructions can not be satisfied especially in patients with infections and weak healing ability [38]. On the way searching for new material strategies that can overcome these limitations, a few studies have shown that graphene, without signs of cytotoxicity, accelerated the proliferation and differentiation of human mesenchymal stem cells (hMSCs) into bone cells with a rate that is comparable to the one achieved with common growth factors [44]. Starting from these results, the possible roles of graphene in enhancing osteogenesis have been extensively investigated.

Among graphene derivatives, GO, with many functional groups, has outstanding surface activities which can exert adsorptive capability to drugs, growth factors, and other biomolecules. Several in vitro experiments have demonstrated that pristine GO may upregulate *β*-catenin protein expression and activate catenin/Wnt signaling pathway, markedly increasing the degree of proliferation and differentiation of cultured cells, and led to acceleration of bone formation [45, 46]. A study conducted by Nishida et al. evaluated the tissue proliferative behaviors in relation to GO scaffold in the tooth extraction socket of dogs. It was observed that the bone formation in GO scaffold was fivefold more than collagen scaffold, which further confirmed the high bone-forming capability of GO scaffolds [47].

Besides being used in their pristine form, they can be combined with different biomaterials. The resultant graphene modified scaffold presented enhanced bioactivity. Addition of GO to chitosan 3D scaffold's composition stimulated the interconnected pore structure, improved mechanical properties, and enhanced the bioactivity of the scaffold materials for osteogenesis [48]. *β*-tricalcium phosphate scaffold modified with GO significantly stimulated the proliferation and osteogenic differentiation of human bone marrow stromal osteoprogenitor cells and, importantly, accelerated new bone formation* in vivo* [16]. GO application to 3D collagen scaffolds stimulated tissue ingrowth behaviors improved their physical properties, enzyme resistance, and Ca and proteins absorption [45].

Dental pulp stem cells (DPSC) are self-renewing and multipotent cells which contain mesenchymal stem cells that can relatively easily obtained from the extracted teeth without esthetic damage [49]. They may undergo both osteoblastic and odontoblastic differentiation making it an interesting model for tissue regeneration. Rosa et al. first confirmed the capability of GO to allow DPSC attachment and proliferation [50]. Xie et al. then tested the potential ability of graphene to induce DPSC's odontogenic and osteogenic differentiation. Without stimuli from bioactive factors, graphene produced by the chemical vapor deposition (CVD) method downregulated the expression of odontoblastic genes (MSX-1, PAX, and DMP), which implied that CVD grown graphene may not serve as a platform for endodontic and pulp regeneration. However, osteogenic genes and proteins including RUNX2, COL, and OCN were significantly upregulated on graphene. This is very likely that the osteogenic differentiation of stem cells presented higher potential as the rigidity of substrate increased; conversely, the odontogenic differentiation of DPSC is better achieved on soft substrate. Nevertheless, graphene could be a potential material to be used for bone tissue engineering and regeneration.

Another stem cell from dental tissue that can be influenced by graphene scaffold is periodontal ligament stem cells (PDLSCs). They could be obtained from dental tissue and have shown multiple differentiation capacity and immunomodulation, capable of differentiating into both cementoblasts and collagen forming cells [51]. A study conducted by Xie et al. investigated the expression profile of PDLSCs for osteogenic differentiation on two-dimensional graphene (2DGp) and three-dimensional graphene scaffold (3DGp) [52]. The result showed that, in both arrangements, without use of osteogenic medium, there was an increase in bone related genes RUNX2 and OCN. Remarkably, an upregulation of MYH10 and MYH10-V2 was observed, especially in 3DGp. MYH10 and MYH10-V2 are cytoskeletal proteins associated with mitochondrial DNA which increase with substrate rigidity. Their upregulation implied that substrate stiffness may play a role in regulating PDLSCs cell lineage specification and promoting differentiation. In fact, it was previously established by other researches that compared to two-dimensional structure; three-dimensional scaffold allowed stem cells to respond better to hormones and exhibited lower requirements for growth factor, thereby improving stem cells' viability, degree of efficiency, consistency, and predictability [53, 54]. Nevertheless, this study suggested that both chemical characteristics and intrinsic physical properties of graphene take part in osteoblastic differentiation of PDLSCs [52].

Research has also been done to analyze the performance of Silk fibroin (SF) and GO in cell proliferation and mesenchymal phenotype expression of PDLSCs [55]. Although SF was previously proved to allow optimal adhesion of mesenchymal stem cell, PDLSCs showed limited attachment on it. When GO was added to SF, layered molecular structures of fibroin and graphene reinforced each other producing an unusually high robust construction, very suitable to serve as a cellular environment where mechanical resistance is required. As it was shown under MTT assay and cytometry, without interfering the mesenchymal phenotype of PDLSCs, the initial adhesion of PDLSCs was significantly improved and rapid spreading was observed. This fact opens a big step for usage of graphene in regenerative dentistry. Another study conducted by Sanchez et al. tested the capability of PDLSCs cultured on GO/SF or rGO/rSF composite to initiate cementoblast differentiation. Remarkably, together with enhanced level of RUNX2, ALP, and OSX, there was an overexpression of CEMP1, which is a novel cementum component exclusively expressing for cementoblasts and their progenitors. This finding may suggest the presence of advanced spontaneous cementoblast differentiation with moderate rate of proliferation [56]. This is very appreciated in cell regeneration as most artificial materials require multiple growth factors to promote HSCs differentiation, whereas GO/SF may provide a new stage for cementoblast differentiation in the absence of biochemical factor [57].

Only part of recent studies was included in this section; nevertheless, it could be speculated that graphene is one of the most promising biocompatible scaffolds for MSCs adhesion, proliferation, and differentiation, particularly toward the osteogenic lineage. Graphene alone with optimal concentration may spontaneously drive stem cells' osteogenesis, but this effect could be further promoted with presence of growth factors. For future engineering of graphene-based substrates for targeted biomedical applications, a deeper understanding of the ability of graphene to improve the biological properties of different scaffold materials will be essential for future biomedical applications.

## 5. Potential Applications of Graphene in Dental Implants

After dental implantation, fibroosseous integration took place between host biological system and dental implant. At the hard tissue interface, osteogenic properties of implant material are essential for osseointegration while at the soft tissue interface, to ensure a tight epithelial seal preventing bacterial invasion is obligatory [58]. Leak of seal at either interface may result in bacterial contamination and colonization, which may eventually impair osteogenesis and induce bone loss [59]. Therefore, the challenge today is to outrace bacterial colonization over tissue integration, which may be achieved by either inhibiting microorganism colonization or accelerating tissue healing and osseointegration [60].

Even though the alternative materials for dental implants have gained increasing interests, titanium or titanium alloy still remained popular to be the material of choice [57]. Recent nanotechnology researches have been reporting that modifications on titanium implant features such as surface composition, hydrophilicity, surface roughness topography, and geometry can affect the rate and quality of osseointegration [61]. Due to graphene's potential osteogenic and antibacterial ability, it appeared to be an excellent implant coating material to favor better osseointegration.

When graphene is coated on titanium substrate, the hydrophobic character of graphene film exerted self-cleaning effect on its surfaces decreasing the adhesion of microorganism including* S. sanguinis* and* S. mutans*. Additionally, compared to titanium alone, graphene possesses osteogenic property enhancing the expression of osteogenic related genes RUNX2, COL-I, and ALP, boosting osteocalcin gene and protein expression, and consequently increasing the deposition of mineralized matrix [35]. Attempts had also been made to coat GO on titanium substrate as a cell culture platform for PDLSCs differentiation. GO-Ti substrate provided a suitable environment for the attachment, proliferation, and differentiation of PDLSCs. When compared with Na-Ti substrate, expression level of osteogenesis-related markers of COL-I, ALP, BSP, RUNX2, and OCN was higher [62]. These findings confirmed that coating of titanium with graphene could be a promising strategy to improve osseointegration and prevent biofilm formation on implants and devices.

Multiphase nanocomposite could also be a promising biomedical material to prevent implant-associated infection. As discussed in the previous session, the antibacterial properties of GO had been contradicting; hence a study conducted by Jin et al. added an antimicrobial nanomaterial, silver nanoparticle, to the GO-Ti composite. GO, with many carboxyl, hydroxyl, and carbonyl on its surfaces, are negatively charged, which could readily combine with positively charged Ag ions in the aqueous solution. Loading GO thin film and silver nanoparticles onto titanium exhibited excellent antiadherence and antimicrobial ability, especially toward* S. mutans* and* P. gingivalis* [63].

Hydroxyapatite (HA) coating is widely applied as an osteoinductive modification of titanium implant; however its slow biological interaction mechanism and low mechanical strength restricted its application [64]. Graphene oxide/chitosan/hydroxyapatite-titanium (GO/CS/HA-Ti) is produced by incorporating GO and chitosan (CS) into hydroxyapatite-titanium substrate through electrophoretic deposition method. It showed better bioactivity by improving the adhesion, proliferation, and differentiation of BMSC cells* in vivo* and possessed superior osseointegration* in vitro*. Furthermore, bonding strength between composite coating and titanium substrate of GO/CS/HA composite coating were enhanced compared with HA, GO/HA, and CS/HA coatings [65].

The direct fabrication of graphene on NiTi based dental implant using chemical vapor deposition technique upregulated the expression level of osteogenic related genes (OCN, OPN, BMP-2, and RUNX2) and promoted expression of integrin *β*1 and initial adhesion of MSCs, indicating that graphene-functionalized NiTi allows better MSCs cytoskeleton development and spontaneous osteogenic differentiation [66].

Aside from that, GO can be used as a carrier for BMP-2, an osteoinductive, and Substance P, a MSC recruitment agent. Application of GO on titanium allowed dual delivery of SP and BMP-2, showing the greatest new bone formation on Ti implant in the mouse calvaria [67]. However, BMP-2 has short half-life hindering its long-term release at therapeutic dose. Ren et al. further incorporated GO and rGO into a hydrothermally prepared porous titanate scaffold on Ti implants, constructing a delivery vehicle for dexamethasone, an osteoinductive synthetic glucocorticoid. Their study results showed that both DEX-GO-Ti and DEX-rGO-Ti enhanced ALP activity of rBMSCs and upregulated the gene expression levels of OPN and OCN, confirming their osteopromotive ability to promote proliferation and to accelerate osteogenic differentiation of rBMSCs [68].

Despite all the excitement, developing a universally accepted transfer route of graphene to titanium implants remains to be a challenge as the deposition technique should ensure transfer effectiveness without compromising the surface properties graphene possess. Several transferring methods have been developed in the market such as wet method, electrochemical delimitation, thermal release tape, hot press transfer, and roll to roll; these methods may allow large scale transfer of graphene onto planar substrate, but are poorly suited for transferring onto 3D objects such as dental implant. Recently, Morin et al. described a vacuum-assisted dry transfer method which is to coat graphene onto the target object and maintained by a mound to sustain conformation. Using a pressure differential provided via partial vacuum, uniform force is applied to the surface facilitating successful coating of graphene onto the object without changing graphene characteristics [69].

Graphene and its derivatives when coated on titanium implant have remarkable abilities to improve properties of titanium, enabling binding of biomolecules, and induce osseointegration. These characteristics place them under the spotlight for improvement and modification of implant materials.

## 6. Potential Applications of Graphene in Endodontics

The purpose of root canal treatment is to biomechanical clean an infected root canal system to destroy intracanal pathogens, decontaminate residually infected tissue, and avoid further intraoperative/postoperative infection. The main cause of endodontic failure is persisting infection in the root canal [70]. Recently developed photodynamic therapy has gained attention for effective canal disinfection while preserving dentin structures. One of the material that plays a key role in this technique is nontoxic photosensor, such as indocyanine green (ICG), but its poor stability and concentration-dependent aggregation have been concerning [71]. Modifying ICG with GO not only significantly reduced number of* E. faecalis* and* S. mutans*, but also improved the stability and bioavailability of ICG, preventing its degradation and aggregation. [72].

For many years, sodium hypochlorite has been used as the most common intracanal irrigants for its strong antibacterial and tissue-dissolving abilities. However, sodium hypochlorite extrusion during root canal treatment causes acute immediate symptoms and serious potential sequelae including rapid hemolysis and ulceration of surrounding tissues, destruction of endothelial, and fibroblast cells [73]. Incorporating graphene into silver nanoparticles showed strong antibacterial property, as efficacy as 3% sodium hypochlorite in canal disinfection, but with less cytotoxic effect to bone and soft tissues [74].

Bioactive cements have been widely used in endodontics for management of perforation, retrograde root filling, and pulp capping. Among them, Biodentine (BIO) and Endocem-Zr (ECZ) are considered as the safest cements that exhibits the least discoloration and calcification of tooth, but with shortcomings such as high pull-out bond strength, long setting time, and modest mechanical properties. With 3 wt % addition of graphene nanosheets, the setting time of both cements significantly decreased, which could be explained by the role of carbon based materials to act as a matrix for the development of C–S–H and calcium hydroxide, thereby reducing the induction period and accelerating the hydration process [75]. However, a decrease in push out strength of ECZ was observed which requires further studies for clinical use.

Another cement, calcium silicate (CS), well known as mineral trioxide aggregate (MTA), has become popular in endodontic treatment in recent years. It is a hydration product of Portland cement, which has the capacity to upregulate differentiation of odontoblasts and promote calcium phosphate deposition [76]. However, its brittle nature, low fracture toughness, and poor wear resistance, because of the presence of relatively large pores that are potential in initiating macrocracks, have been hindering its use in clinical application. Thus, attempt was made to use GFNs as reinforcement to CS to enhance their mechanical properties. With incorporation of GFNs, significant grain size refinement occurs as GFNs may wrap around grains and inhibit their growth. Aside from decrease in grain size, through crack bridging, pull-out GFNs, crack branching, and crack deflection mechanism, there was also an increase in indentation fracture toughness and brittle index. Moreover, incorporation of GFNs into CS has a beneficial effect on proliferation of human osteoblastic cells (hFOB), indicating their biocompatibility suitability for cell proliferation [77].

Incorporating GO into Portland cement also exhibited positive influence on the workability of the cement. It enhanced the degree of hydration by increasing the nonevaporable water content and calcium hydroxide hydroxide content, contributing to the refinement of pore structures, and subsequently increasing the compressive strengths and suppressing crack propagation in the matrix at nanoscale. Introduction of 0.03% by weight GO into cement paste can increase their compressive strength and tensile strength by more than 40% [78].

In conclusion, addition of graphene into dental cements leads to refinement of pore structure which not only strengthens the cement material but also blocks the entry for possible bacterial invasion. It could be promising reinforcing cementitious material for future dental applications.

## 7. Potential Applications of Graphene in Restorative Dentistry

Glass ionomer (GI), with favorable coefficient of linear thermal expansion, ability to chemically bond to sound tooth structure, and dynamic fluoride release, had been utilized in a wide range of clinical application. However, its poor physiomechanical properties remained to be a concern despite the developments in GI constituents with addition of various filler types including fibers, metallic powders, and hydroxyapatite powders [79]. In recent years, attempts had also been made to incorporate graphene derived nanomaterial into commercially available glass ionomer for reinforcement. Graphene, when combined with glass ionomer prepared with poly(acrylic acid), has significantly enhanced physiomechanical properties of GIs [11]. Fluoride graphene when prepared by hydrothermal reaction of graphene oxide and mechanically blend with glass ionomer could produce a GICs/FG composites matrix, which could significantly enhance the mechanical, tribological, and antibacterial properties of glass ionomer [80]. With the increase of FG content in glass ionomer, there is a decrease of pores and microcracks in the internal structure of material and an increase in antibacterial ability making it less susceptible to erosion disintegration and microbial invasion. Reinforcing resin polymer matrices with graphene gold nanoparticles as fillers showed improvement of degree of conversion and surface properties, offering a good solution to improve physicochemical properties of dental nanocomposite [81].

Due to the presence of microcavities between the healthy tissue and dental restoration causing bacteria invasion, there has been an increasing interest in development of antibiofilm adhesives. One concern with dental adhesive monomers is its excessive ROS production which not only cause oxidative stress associated toxicity toward fibroblasts and pulp cell, but also affect saliva redox equilibrium and decreasing natural oral immune system defenses [82]. Due to graphene's antibacterial properties, Bregnocchi et al. proposed using GFNs as an antimicrobial and antibiofilm filler for dental adhesive. The study result showed that GFNs modified dental adhesive significantly inhibited the adhesion and growth of* S. mutans* without interfering its original mechanical performances and without producing a surplus of ROS [83].

## 8. Potential Applications of Graphene in Periodontology

In treatment for periodontal bone defects using guided tissue regeneration (GTR) and guided bone regeneration (GBR), barrier membranes have been a crucial biomaterials, which is to create a secluded space between soft connective tissue and regenerating bone, for formation of unimpeded bone promoting faster differentiation of mesenchymal cells into odontoblast/osteoblast [84]. Attempts to modify barrier membrane have been made to improve its biocompatibility. Radunovic et al. investigated the effect of collagen membrane coated with GO (10 *μ*g/mL) on the viability and metabolic activity of dental pulp mesenchymal cells. The result showed that GO coating at the higher concentration induces PGE2 secretion, controls inflammation, and promotes DPSCs differentiation which is probably due to GO's large reactive surface area providing idea platform for biofunctionalization and concentrating chemical, proteins, and growth factors for faster differentiation [85]. An animal study conducted by Kawamoto evaluated the periodontal wound healing capability of GO scaffold on dogs with class II furcation defect. The result showed that GO scaffold succeeded in periodontal ligament like and cementum like tissue formation, followed by alveolar bone formation. The study also speculated that GO application to collagen scaffold stimulated scaffold degradation and replaced them with newly regenerated periodontal tissue [86].

## 9. Conclusion

**Figure 3:**
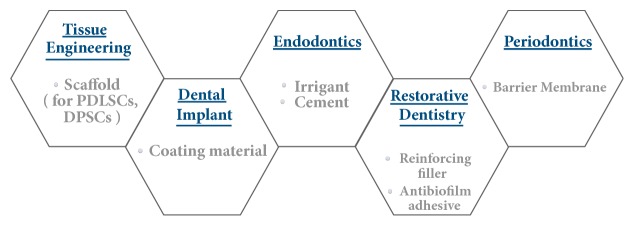
Currently, no studies correlating dentistry and GFNs have been done on human subjects. Based on the properties presented in* in vivo* and* in vitro* studies, the potential applications of GFNs that could be translate to clinical reality in dentistry were summarized by different dental disciplines.

Researches toward graphene in dental materials mainly focused on two ways: one is to prepare new dental materials as GFNs alone, and the other is to modify the common dental materials by transferring appropriate GFNs onto different substrates. As clearly highlighted in this paper, in either way, GFNs improved the physical, chemical, and mechanical properties of biomaterials, holding enormous potential in new therapeutic strategies in dental field. In this review, we firstly discussed the toxicity and antibacterial properties of GFNs on cells* in vitro* and* in vivo.* Then, we presented a comprehensive summary of the latest research progress related to the potential applications of GFNs in dentistry (see Figure 3).

Safety and potential risks of GFNs should be emphasized and research efforts should be made to ensure harmless use of graphene in oral environment. Additionally, some very promising properties of GFNs have been extensively investigated in other organs of human body* in vitro* and* in vivo*, but no studies have been done on human subjects and in-depth studies are still scarce in oral settings. GFNs is a rather fascinating material worthy of in-depth investigation; further exploration on their underlying mechanism of interaction toward oral tissues in terms of cell-signaling, metabolic pathway, osteogenesis, and antibacterial effects is needed. We hope that this review article could provide some valuable elicitation for the future scientific and technological innovations of graphene in dentistry.
